# Repeated Use of the Psychoactive Substance Ethylphenidate Impacts Neurochemistry and Reward Learning in Adolescent Male and Female Mice

**DOI:** 10.3389/fnins.2019.00124

**Published:** 2019-02-19

**Authors:** Meridith T. Robins, Arryn T. Blaine, Jiwon E. Ha, Amy L. Brewster, Richard M. van Rijn

**Affiliations:** ^1^Department of Medicinal Chemistry and Molecular Pharmacology, Purdue University, West Lafayette, IN, United States; ^2^Purdue Institute for Integrative Neuroscience, Purdue University, West Lafayette, IN, United States; ^3^Purdue Interdisciplinary Life Sciences Graduate Program, Purdue University, West Lafayette, IN, United States; ^4^Department of Psychological Sciences, Purdue University, West Lafayette, IN, United States

**Keywords:** ethylphenidate, novel psychoactive substance, adolescence, BDNF, deltaFosB, conditioned place preference, Barnes maze

## Abstract

Schedule II prescription psychostimulants, such as methylphenidate (MPH), can be misused as nootropic drugs, i.e., drugs that enhance focus and cognition. When users are unable to obtain these prescribed medications, they may seek out novel psychoactive substances (NPSs) that are not yet scheduled. An example of a NPS reportedly being abused is ethylphenidate (EPH), a close analog of MPH but with a higher preference for the dopamine transporter compared with the norepinephrine transporter. Therefore, based upon this pharmacological profile and user self-reports, we hypothesized that repeated EPH exposure in adolescent mice may be rewarding and alter cognition. Here, we report that repeated exposure to 15 mg/kg EPH decreased spatial cognitive performance as assessed by the Barnes maze spatial learning task in adolescent male C57Bl/6 mice; however, male mice did not show alterations in the expression of mature BDNF – a protein associated with increased cognitive function – in key brain regions. Acute EPH exposure induced hyperlocomotion at a high dose (15 mg/kg, i.p.), but not a low dose (5 mg/kg, i.p.). Interestingly, mice exhibited significant conditioned place preference at the low EPH dose, suggesting that even non-stimulating doses of EPH are rewarding. In both males and females, repeated EPH exposure increased expression of deltaFosB – a marker associated with increased risk of drug abuse – in the dorsal striatum, nucleus accumbens, and prefrontal cortex. Overall, our results suggest that repeated EPH use in adolescence is psychostimulatory, rewarding, increases crucial brain markers of reward-related behaviors, and may negatively impact spatial performance.

## Introduction

The past decade has seen a rampant rise in the reported number of NPSs – also known as “legal highs” (Smith et al., 2015). NPSs, which are either novel in origin or novel for the indication or purpose of their use (Schifano et al., 2015), can alter the mental and behavioral performance of the user; however, the risks of acute or repeated use are largely unknown (Hassan et al., 2017). Indeed, some of these NPSs may be toxic upon consumption or lead to significant modification of mental status after intake (McKenna, 2004; Hagen et al., 2009; Soussan and Kjellgren, 2015; Maskell et al., 2016). Because of the rapid proliferation of NPSs, NPSs are now illegal to distribute or sell in countries such as the United Kingdom (however, these substances are still legal to possess in the United Kingdom), while in other countries, NPSs remain unregulated (Tracy et al., 2017). NPS use is most commonly associated with young adult males approximately 18 years of age (middle to late adolescence) (Vardakou et al., 2011; Werse and Morgenstern, 2012), although use is also reported in adults, suggesting that NPS use is not only a youth phenomenon. NPS use widely varies between countries (European Monitoring Centre For Drugs And Drug Addiction, 2017), although lifetime prevalence is estimated to be 4% and last year prevalence to be 3% for European students. As for prevalence, NPSs use is much smaller than that of substances illegal to adolescents, such as alcohol, nicotine, or THC.

While some NPSs can be categorized as cannabinoids, hallucinogenics, or depressants (Tracy et al., 2017; Evans-Brown and Sedefov, 2018), many NPSs act as psychostimulants. Psychostimulant NPSs include synthetic cathinones, (Luethi et al., 2017) and EPH, a compound similar in structure and function to the attention-deficit hyperactivity -disorder (ADHD) medication MPH (Ritalin^®^). EPH initially rose in prominence in Europe in the past decade under the name of “diet coke” or “nopaine” (Bailey et al., 2015; Parks et al., 2015). Self-reports of EPH use from internet forums found a median age of 23 for those using EPH (with a range from 19 to 42) (Soussan and Kjellgren, 2015). In humans, self-reports of EPH consumption are associated with a number of altered behaviors including increased socialness, euphoria, cognitive enhancement, as well as bodily agitation, insomnia, anxiety, and compulsive use (Ho et al., 2015; Soussan and Kjellgren, 2015). Moreover, EPH use has also been associated with weight loss, irritability, and paranoia along with potential long-term mental health disorders (Lafferty et al., 2016; Robertson, 2017). As a result of the increased reports of EPH use and toxicity (Maskell et al., 2016), EPH became illegal to manufacture, sell, or import in many European countries starting in 2012. As of 2018, EPH is not explicitly a controlled substance in the United States; however, because it is an analog of MPH, which is a Schedule II substance, EPH would be classified as a Schedule II substance if sold with the intent for human consumption (World Health Organization, 2016).

For the majority of NPSs, detailed pharmacological data is not available; however, *in vitro* and *in vivo* pharmacology studies have found that EPH is similar to MPH and cocaine in its mechanism of action (Patrick et al., 2005; Williard et al., 2007; Luethi et al., 2017; Davidson et al., 2018). EPH stimulates locomotor activity in mice at 5 and 10 mg/kg (±)-EPH in C57Bl/6 mice (Williard et al., 2007). In HEK293 cells expressing human DAT, racemic (±)-EPH has increased potency for DAT inhibition (95 ± 18 nM) compared to cocaine (289 ± 38 nM). The ability of EPH to inhibit DAT is primarily driven by (+)-EPH, with DAT inhibition at 26 ± 6 nM, versus (-)-EPH with 1730 ± 180 nM DAT inhibition (Patrick et al., 2005). Negligible binding and inhibition is observed at the SERT for (±)-EPH, while similar NET inhibition and binding is detected between cocaine and (±)-EPH. Compared with (±)-MPH, (±)-EPH also displays a higher preference for DAT versus NET in terms of inhibition (2.6- vs. 5.1-fold) and binding (6.5- vs. >22-fold) in HEK 293 cells (Patrick et al., 2005).

In humans, an increased DAT preference for psychostimulants over NET or SERT is commonly correlated with psychotropic effects (Simmler et al., 2013), a notion in agreement with the reports of euphoria in human users of EPH (Soussan and Kjellgren, 2015). Another DAT preferring stimulant, 3,4-methylenedioxypyrovalerone, produces CPP at much lower dose than amphetamine in C57Bl/6 mice (Simmler et al., 2013; Karlsson et al., 2014) and produces cognitive deficits upon repeated exposure in rats (Sewalia et al., 2018). Additionally, DAT KO mice have been shown to display poor Morris water maze performance (Morice et al., 2007; Weiss et al., 2007). Based on the stated reports indicating a role for DAT in reward and cognitive processes, we hypothesized that EPH, because it has increased DAT preference, would be stimulatory, induce place preference and give rise to cognitive deficits upon prolonged exposure.

To test our hypothesis, we determined how exposure to EPH in adolescent male and female C57BL/6 mice affected cognitive outcomes, as evaluated through the Barnes maze. In parallel, we determined the levels of brain expression of BDNF, a protein frequently associated with the modulation of memory and cognitive processes (Savitz et al., 2006; Lu et al., 2014; Menard et al., 2015; Kowianski et al., 2018). We determined the stimulatory and rewarding properties of EPH by measuring general locomotor activity, locomotor sensitization, and CPP to high (15 mg/kg) and low doses (5 mg/kg) of EPH. The expression of ΔFosB in mesocorticolimbic brain regions was used to assess repeated activation of areas associated with drug addiction (Kelz et al., 1999; Nestler et al., 2001; Perrotti et al., 2008).

## Materials and Methods

### Drugs and Chemicals

(±)-threo-ethylphenidate hydrochloride (EPH) was purchased from Cayman Chemical (Ann Arbor, MI, United States). Ketamine was purchased from Henry Schein Animal Health (Dublin, OH, United States) and xylazine and heparin (10 units/mL) from Sigma-Aldrich (St. Louis, MO, United States). Paraformaldehyde ampules were obtained from Electron Microscopy Sciences (Hatfield, PA, United States).

### Animal Husbandry

**Figure 1:**
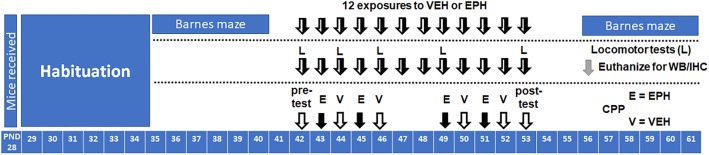
Timelines of experiments performed. Adolescent male and female C57Bl/6 mice (*n* = 6/group) were trained on the Barnes maze for 1 week prior [postnatal day (PND)35–40] to 12 days of vehicle (VEH, V) or ethylphenidate (EPH, E) exposure (PND42–53), followed by a week of post-drug Barnes maze training (PND56–61). For locomotor, Western blot (WB), and immunohistochemistry (IHC) studies, mice were exposed to 12 consecutive days of vehicle or drug exposure (PND42–53), where locomotor testing (L) was conducted on days 1, 3, 5, and 12 of drug exposure (euthanized PND56). A 2-week conditioned place preference protocol (CPP) was performed, with one pre-test (PND42), 8 days of conditioning to either vehicle or drug (4 days each, PND43–53), and 1 day of post-testing (PND53).

Male and female C57Bl/6, wild-type adolescent (postnatal day 28) mice were purchased from Envigo (Indianapolis, IN, United States) and habituated for 1 week to the animal facility prior to behavioral testing. Food and water was provided *ad libitum*. Throughout the experiment, animals were kept at ambient temperature of (21°C) in a room maintained on a 12L:12D cycle (lights on at 9.00, lights off at 21.00) in Purdue University’s animal facility, as accredited by the Association for Assessment and Accreditation of Laboratory Animal Care. All animal procedures were pre-approved by Purdue University’s Institutional Animal Care and Use Committee (#1605001407) and conducted in accordance with National Institutes of Health Guide for the Care and Use of Laboratory Animals. A general timeline of the experiments performed is shown in Figure 1.

### Barnes Maze Task for Spatial Learning

The Barnes maze task for spatial learning was initially conducted in drug-naïve male and female adolescent mice as described previously (Sunyer et al., 2007; Patil et al., 2009) over the course of 6 days in a light room with bright light and geometric shapes taped to the walls for spatial cueing. On day 1, mice were habituated to the maze’s escape route over the course of three sessions. On days 2–5, four trials a day, at least 15 min apart in time were conducted to assess latency to enter the escape route. Each trial ended once the mouse entered the escape route or after 3 min (if the animal did not enter the escape route before the 3-min session was finished, the animal was guided to the escape route and placed inside). Following each trial, the animal was left in the escape route for 1 min.

Following the initial Barnes maze training, animals were exposed to once daily injections of 15 mg/kg EPH (i.p.) over a period of 12 consecutive days. Three days after the final EPH injection, Barnes maze re-testing began as described previously (days 1–5 described above). Total time to enter escape route per trial and total errors per trial per recorded manually by an unbiased experimenter. All testing was conducted during the animals’ light cycle, and animals were habituated to the testing room for at least 1 h prior to testing and conditioning sessions. The maze was cleaned with 70% isopropyl alcohol between animal trials to prevent lingering scent trials.

### Western Blot for BDNF Expression in the Prefrontal Cortex, Cortex, and Hippocampus

Mice were euthanized via CO_2_ asphyxiation prior to brain dissection. Specific brain regions (cerebellum, cortex, hippocampus, prefrontal cortex) were stored on dry ice. Whole tissue samples were lysed using 200 μL (hippocampus) or 300 μL (cerebellum, cortex, prefrontal cortex) of RIPA buffer and 1x protease inhibitor (Thermo Fisher, Waltham, MA, United States). Samples were then homogenized by repeated passage through a 1 mL BD syringe with 25-gauge needle (Thermo Fisher), followed by sonication. Samples were stored on ice briefly and centrifuged at 12,000 × *g* for 20 min at 4°C. Following centrifugation, the supernatant was kept for further analysis. Protein concentration was determined by Bradford protein determination assay (Bio-Rad, Hercules, CA, United States). Samples were then loaded at 20 μg protein/20 μL into a NuPAge 4–12% Bis-Tris gradient gel. The gel was then transferred to a nitrocellulose membrane (Thermo Fisher) for blocking (Li-Cor Blocking Buffer, Lincoln, NE, United States). Following blocking, the membrane was stained with two primary antibodies: rabbit anti-BDNF (1:1000, ab108319, Lot GR321753-21, Abcam, Cambridge, MA, United States) and mouse anti-α-tubulin (1:2000, sc-5286, Lot G3117, Santa Cruz Biotechnology, Dallas, TX, United States) in blocking buffer consisting of 0.2% Tween-20 for 1 h at room temperature. After washing, the membranes were stained with secondary antibodies IRdye 800 CW goat anti-rabbit (926-32211, Lot C61103-06, Li-Cor) and IRdye 680 LT got anti-mouse (925-68020, Lot C60824-02, Li-Cor) at a dilution of 1:5000 in blocking buffer for 1 h at room temperature while rocking.

Membrane imaging was performed using a Li-Cor Odyssey CLx. All blots (proBDNF, BDNF, or α-tubulin) were imaged at the same intensity for proper comparison between subjects, sexes, and brain regions. The relative density of the immunoreactive bands for proBDNF (32 kDa) and mature BDNF (14 kDa) were determined through normalization to α-tubulin (loading control in same lane) and subtracting background using ImageJ software (NIH, Bethesda, MA, United States) as previously described (Schartz et al., 2018).

### Acute and Repeated Exposure Locomotor Activity

On day 1 (first day of drug exposure), animals were weighed and injected with vehicle, 5, or 15 mg/kg EPH i.p. directly prior to a 60-min locomotor session in square locomotor boxes from Med Associates (L 27.3 cm × W 27.3 cm × H 20.3 cm, St. Albans, VT, United States). Locomotor testing was conducted on days 1, 3, 5, 8, and 12 following drug exposures; on days without locomotor testing, animals were injected with vehicle or EPH and placed back in their home cage. All testing and drug administration was conducted during the animals’ light cycle, and animals were habituated to the testing room for at least 1 h prior to testing sessions.

### Conditioned Place Preference

An unbiased CPP protocol was performed as previously described (Cunningham et al., 2006; Robins et al., 2016). In brief, adolescent male and female animals were placed in a two-chamber apparatus for 30 min following a vehicle injection (0.9% saline, i.p.) to establish initial bias. Animals exhibiting an initial bias for one compartment >70% were excluded from further testing. Over the following conditioning days, one conditioning session (30 min) was performed per day by confining the animal to either drug- (5 or 15 mg/kg EPH) or vehicle-paired side for a total of eight conditioning sessions. On the final day, animals were placed in the two-chamber apparatus following a vehicle injection to freely explore both compartments where preference of the two chambers was assessed over 30 min. All testing was conducted during the animals’ light cycle, and animals were habituated to the testing room for at least 1 h prior to testing and conditioning sessions.

### Immunohistochemistry

Male and female adolescent mice were exposed to daily vehicle, 5 mg/kg, or 15 mg/kg EPH (i.p.) injections for 12 consecutive days. Three days following the final drug administration, animals were transcardially perfused as previously described (Robins et al., 2016). Brains were fixed in a 4% paraformaldehyde solution for 24 h before transfer into 30% sterile sucrose (Sigma) for at least 1 week for cryoprotection. The sucrose solution was changed once during this time. Brains were embedded and frozen in Tissue-Tek^®^ O.C.T. compound (VWR, Radnor, PA, United States) in tissue molds (VWR) and 50 μm coronal sections were prepared using a cryostat (Leica Microsystems Inc., Buffalo Grove, IL, United States). Staining was conducted on free-floating slices for ΔFosB positive cells using primary goat anti-ΔFosB antibody (sc-48-G, Santa Cruz Biotechnology, Dallas, TX, United States), diluted 1:1000, and secondary Alexa-Fluor 594 donkey anti-goat antibody (A-11058, Life Technologies, Grand Island, NY, United States), diluted 1:1000. Slices were mounted with VectaShield (Vector Laboratories, Burlingame, CA, United States) mounting media on microscope slides (Fischer Scientific, Hampton, NH, United States), fitted with coverglass (Fischer Scientific), and sealed with nail polish.

Images were acquired via confocal microscopy (Nikon A1) at 20× magnification using an oil immersion objective. Gain and exposure were standardized to slices from a vehicle-treated animal for proper control throughout image capture. For each animal, two images were collected, one image from the left hemisphere and one from the right hemisphere for the brain region of interest. Images were processed using ImageJ software (National Institutes of Health) for the number of ΔFosB positive cells in the dorsal striatum, shell of the nucleus accumbens, or prefrontal cortex (prelimbic cortex) per image. Positive cells were identified as areas with a specific intensity and area compared to background, as identified through ImageJ analysis. The total area of analysis for each images = 0.403 mm^2^.

### Statistical Analysis

All data are presented as means ± standard error of the mean and analysis was performed using GraphPad Prism 8 software (GraphPad Software, La Jolla, CA, United States). Two-way, repeated measures ANOVA with Bonferroni multiple comparisons test was used for locomotor differences between first and last drug exposure and CPP studies. Two-way ANOVA with *post hoc* Tukey multiple comparisons test were utilized to assess drug, sex, or interaction effect for Barnes maze performance, proBDNF and mature BDNF expression via Western blot, locomotor differences at first drug exposure, last drug exposure, difference in time spent on the EPH-paired side in CPP, and ΔFosB accumulation. When assessing sex differences across multiple factors, three-way ANOVA was performed with the factors: sex, treatment, time of testing (Barnes maze, CPP).

## Results

### Mice Acutely Exposed to 15 mg/kg EPH, but Not 5 mg/kg, Display Hyperlocomotion

**Figure 2:**
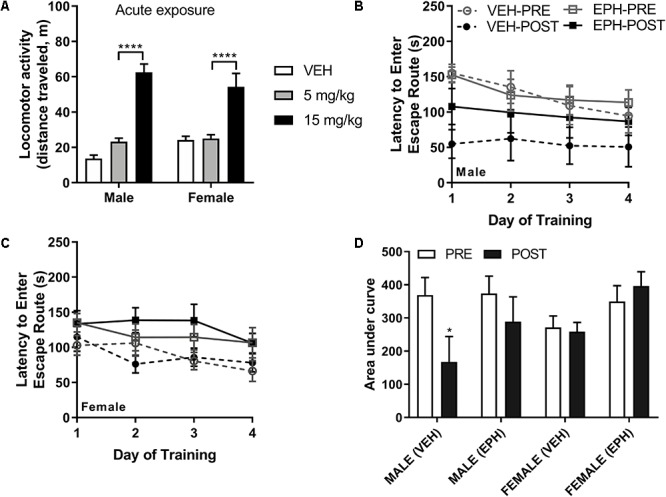
Higher dose of 15 mg/kg EPH is stimulatory and EPH exposure impairs retrieval of escape route memory. Acute exposure to higher 15 mg/kg dose of EPH induced hyperlocomotion in male and female adolescent mice compared with 5 mg/kg EPH or vehicle **(A)**. In a separate cohort of mice, animals were trained on the Barnes maze for 1 week prior to 12 consecutive days of vehicle (VEH) or 15 mg/kg EPH exposure (i.p.), No significant differences in performance were detected prior to drug exposure **(B)**; however, an overall drug effect was in the post-drug testing sessions **(C)**. A decrease in performance was observed upon post-drug Barnes maze testing in male animals exposed to 15 mg/kg EPH compared with males exposed to vehicle **(D)**. Significance by two-way ANOVA with Tukey’s method for multiple comparisons, ^∗^*p* < 0.05; ^∗∗∗∗^*p* < 0.0001; data represented as mean ± SEM.

First, we identified a pharmacologically effective dose of EPH for stimulating locomotion. As EPH is a known psychostimulant (Ho et al., 2015), we tested locomotor activity induced by 5 and 15 mg/kg EPH in adolescent male and female C57BL/6 mice (*n* = 5–6 per group). Here, we found that 15 mg/kg EPH effectively produced hyperlocomotion (Figure 2A). No sex (*F*_1,28_ = 0.178, *p* = 0.676) or interaction (*F*_2,28_ = 3.15, *p* = 0.0581) effect was observed for total ambulation following acute drug exposure, although a significant effect of dose was noted (*F*_2,28_ = 64.0, *p* < 0.0001). In male and female mice, significantly higher ambulation was observed between VEH and 15 mg/kg EPH (*p* < 0.0001) and between 5 and 15 mg/kg EPH (*p* < 0.0001) (Figure 2A); thus, we used 15 mg/kg EPH as a stimulatory dose of EPH in further behavioral testing.

### Performance of Male Mice in the Spatial Barnes Maze Is Decreased Following Repeated Adolescent EPH Exposure

To study how EPH exposure may impact learning and memory, we chose to use the Barnes maze task (Barnes, 1979) as it is capable of assessing spatial learning without inducing significant stress or anxiety, as compared with the Morris water maze (Harrison et al., 2006). Male and female mice were trained on the Barnes maze to locate an escape route within 3 min over the course of 4 days of training (with four sessions per day). All mice showed a decrease in the latency to finding the escape route during training, and following initial training, mice were divided into vehicle or EPH groups with no significant intergroup differences (effect of sex: *F*_1,19_ = 1.67, *p* = 0.212; effect of pre-drug group: *F*_1,19_ = 0.771, *p* = 0.391; interaction effect: *F*_1,19_ = 0.606, *p* = 0.446) (Figure 2B). Following repeated exposure to either 0.9% saline (VEH) or 15 mg/kg EPH (*n* = 6 per group) for 12 consecutive days, mice were again trained on the Barnes maze (Figure 2C). We noted that vehicle treated male mice performed significantly better during the second trial (*p* = 0.019); however, this effect was attenuated in EPH-treated mice. In contrast, female mice did not perform better during the second trial, with no change in performance compared to EPH-treated female mice. These findings were determined by three-way ANOVA with sex (S), drug treatment (D), and time (T) as factors, which revealed significant main effects of time (*F*_1,19_ = 6.66, *p* = 0.018) and sex × time (*F*_1,19_ = 10.74, *p* = 0.004), but not of drug treatment (*F*_1,19_ = 3.36, *p* = 0.08) and sex (*F*_1,19_ = 0.166, *p* = 0.688), or other interactions (Figure 2D). The observed sex differences in Barnes maze performance agrees with prior reports on sex differences in spatial performance in C57Bl/6 mice (O’Leary and Brown, 2012, 2013).

### Male Mice Show Increased proBDNF, but Not Mature BDNF, Expression in Cortex and Cerebellum Following Repeated Adolescent EPH Exposure

Altered levels of BDNF have been observed in the prefrontal cortex, hippocampus, cerebellum, and cortex following psychostimulant exposure, and changes in BDNF levels may be correlated with the therapeutic mechanism of action for ADHD medications and neuronal plasticity (Banerjee et al., 2009; Fumagalli et al., 2010; Scherer et al., 2010; Quintero, 2013; Schmidt et al., 2013; Andersen and Sonntag, 2014; Laricchiuta et al., 2018) as well as spatial learning (Radecki et al., 2005; Kulikov et al., 2014; Petzold et al., 2015). BDNF can be measured as both the immature form, proBDNF, and the cleaved, mature BDNF, where proBDNF is also active and may have unique signaling properties compared to mature BDNF (Hempstead, 2015). Therefore, we quantified changes in both proBDNF and mature BDNF expression in these regions following repeated 15 mg/kg EPH exposure in adolescent male and female mice.

**Figure 3:**
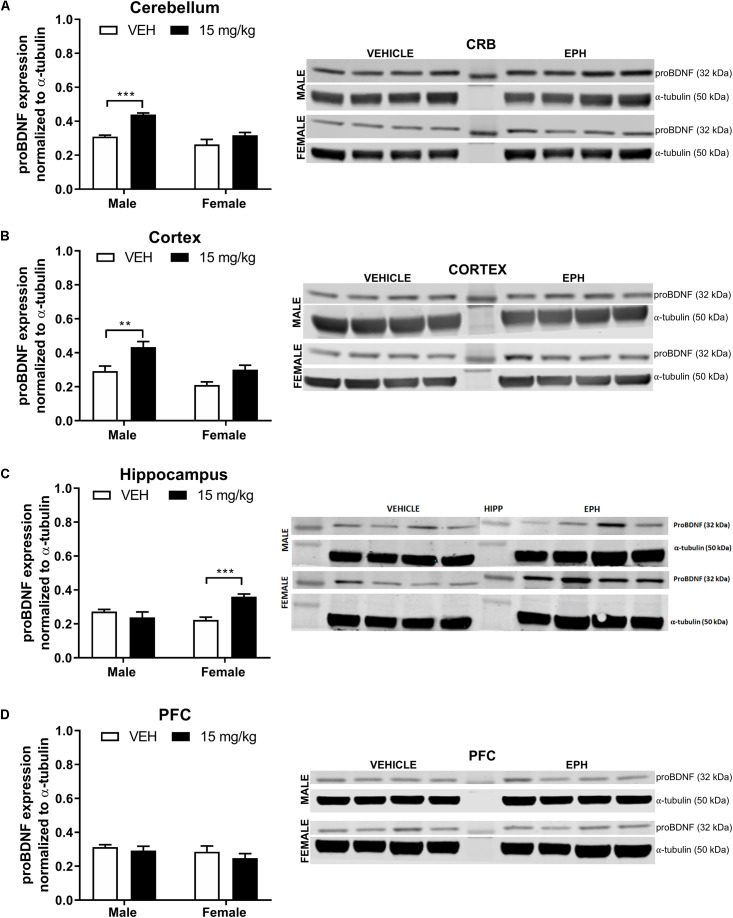
EPH exposure increased proBDNF expression in cerebellar and cortical regions of adolescent male mice. Adolescent male and female C57Bl/6 mice were exposed to a daily intraperitoneal injection of vehicle (VEH) or 15 mg/kg EPH daily for 12 consecutive days. proBDNF expression levels were measured via Western blot for the cerebellum **(A)**, cortex **(B)**, hippocampus **(C)**, and prefrontal cortex **(D)**, where a significant increase in proBDNF expression was found in the cerebellum and cortex of male mice exposed to 15 mg/kg EPH compared with vehicle. Non-immunoreactive bands between vehicle and EPH-treated groups indicate loaded protein ladder. Significance by two-way ANOVA with Tukey’s method for multiple comparisons, ^∗∗^*p* < 0.01; ^∗∗∗^*p* < 0.001; data represented as mean ± SEM.

For proBDNF in the cerebellum (Figure 3A), a significant effect of drug exposure (*F*_1,12_ = 26.5, *p* = 0.0002) and sex (*F*_1,12_ = 22.3, *p* = 0.0005) was observed with no interaction effect (*F*_1,12_ = 4.66, *p* = 0.0519). Multiple comparison analysis revealed a significant increase (*p* = 0.0005) in proBDNF expression in male mice exposed to 15 mg/kg EPH compared with vehicle. Between sexes, male mice exposed to 15 mg/kg EPH exhibited significantly higher proBDNF expression compared with female mice exposed to 15 mg/kg EPH (*p* = 0.0019). Similar results were found in the cortex (Figure 3B), where a significant effect of drug exposure (*F*_1,12_ = 17.7, *p* = 0.0012) and sex (*F*_1,12_ = 15.3, *p* = 0.0021) was observed with no interaction effect (*F*_1,12_ = 0.866, *p* = 0.371). Again, multiple comparisons revealed a significant increase in proBDNF expression in male mice exposed to 15 mg/kg EPH compared with vehicle (*p* = 0.0069). Furthermore, a sex-specific increase was observed in proBDNF expression following 15 mg/kg EPH exposure, with male mice exhibiting higher proBDNF expression than females (*p* = 0.0226). In the hippocampus (Figure 3C), we found a significant effect of drug exposure (*F*_1,12_ = 6.42, *p* = 0.0263) but not sex (*F*_1,12_ = 3.05, *p* = 0.1061) and with an interaction effect (*F*_1,12_ = 17.51, *p* = 0.0013). In contrast to the cerebellum and hippocampus, multiple comparisons revealed a significant increase in proBDNF expression in female mice exposed to 15 mg/kg EPH compared with vehicle (*p* = 0.0009). No significant effects of 15 mg/kg EPH exposure were observed in the prefrontal cortex (Figure 3D, drug: *F*_1,12_ = 1.25, *p* = 0.2857; sex: *F*_1,12_ = 1.91, *p* = 0.192; interaction: *F*_1,12_ = 0.103, *p* = 0.753).

**Figure 4:**
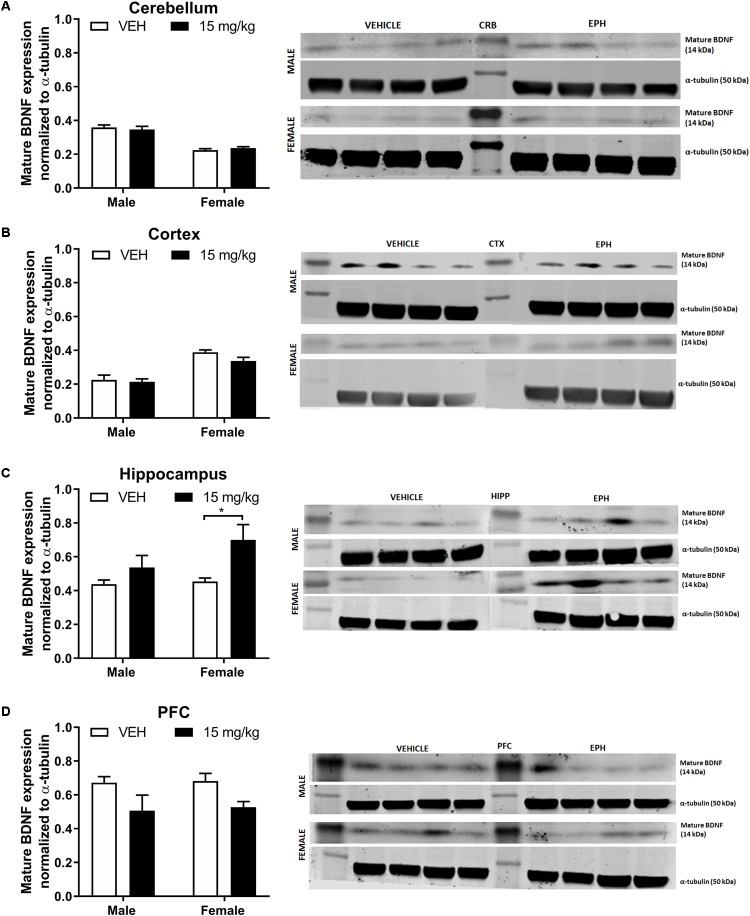
EPH exposure alters mature BDNF expression in cortex and hippocampus. Adolescent male and female C57Bl/6 mice were exposed to a daily intraperitoneal injection of vehicle (VEH) or 15 mg/kg EPH daily for 12 consecutive days. Mature BDNF expression levels were measured via Western blot for the cerebellum **(A)**, cortex **(B)**, hippocampus **(C)**, and prefrontal cortex **(D)**, where a significant increase in BDNF expression was found in the cortex of female mice exposed to EPH compared to male EPH-exposed mice. An overall drug effect was also observed in the hippocampus. Non-immunoreactive bands between vehicle and EPH-treated groups indicate loaded protein ladder. Significance by two-way ANOVA with Tukey’s method for multiple comparisons, ^∗^*p* < 0.05.

Sex appeared to affect mature BDNF expression in the cerebellum (Figure 4A, drug: *F*_1,12_ = 0.0005, *p* = 0.98; sex: *F*_1,12_ = 86.71, *p* < 0.0001; interaction: *F*_1,12_ = 0.77, *p* = 0.397). and the cortex (Figure 4B drug: *F*_1,12_ = 2.29, *p* = 0.156; sex: *F*_1,12_ = 46.77, *p* < 0.0001; interaction: *F*_1,12_ = 0.92, *p* = 0.356),. In the hippocampus (Figure 4C, drug: *F*_1,12_ = 8.37, *p* = 0.0135; sex: *F*_1,12_ = 2.25, *p* = 0.159; interaction: *F*_1,12_ = 1.51, *p* = 0.242), multiple comparisons revealed a significant increase in mature BDNF expression in female EPH-exposed mice compared with vehicle-exposed female mice (*p* = 0.0258). An overall drug effect was observed in the prefrontal cortex (Figure 4D drug: *F*_1,12_ = 7.89, *p* = 0.0158; sex: *F*_1,12_ = 0.07, *p* = 0.798; interaction: *F*_1,12_ = 0.01, *p* = 0.931) for mature BDNF expression.

### Repeated EPH Exposure Does Not Induce Locomotor Sensitization

**Figure 5:**
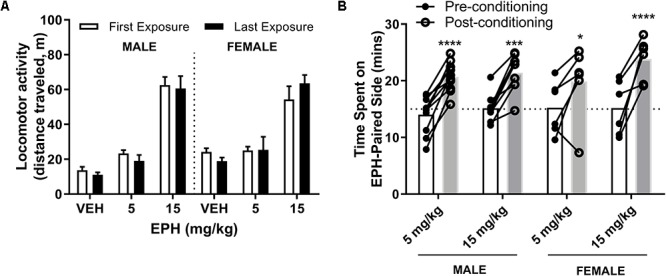
No changes in locomotor observed at 5 or 15 mg/kg EPH upon repeated exposure, but conditioned preference at both non-stimulatory and stimulatory doses. No alterations in locomotor activity were observed between the first or last exposure to EPH at 5 or 15 mg/kg in adolescent male or female C57Bl/6 mice, suggesting no locomotor sensitization upon repeated exposure **(A)**. In conditioned place preference testing, male and female adolescent C57Bl/6 mice spent more time on the EPH (EPH)-paired compartment in a two-chamber, conditioned place preference protocol following eight total conditioning sessions **(B)**. No significant change in time spent on the EPH-paired side by dose or sex. Significance by two-way ANOVA with Tukey’s method for multiple comparisons, ^∗^*p* < 0.05; ^∗∗∗^*p* < 0.001; ^∗∗∗∗^*p* < 0.0001; data represented as mean ± SEM.

Locomotor sensitization is a sign of synaptic plasticity and has been correlated with DAT activity (Fukushima et al., 2007). Here, we assessed locomotor activity following both acute and repeated exposure to EPH at a low (5 mg/kg) or high (15 mg/kg) dose. We did not observe an increase in total ambulatory distance in adolescent male or female mice (*n* = 5–6 per group) at 5 or 15 mg/kg EPH between day 1 (first drug exposure) and day 12 (last drug exposure) (Figure 5A), suggestive of an absence of locomotor sensitization. No significant effect of drug dose × exposure date (*F*_2,28_ = 0.71, *p* = 0.62) or effect of exposure date (*F*_1,28_ = 0.09, *p* = 0.77) was found, while a significant effect of drug dose (*F*_5,28_ = 47.98, *p* < 0.0001) was observed by two-way ANOVA. Additionally, no effect of matching *F*_28,28_ = 1.03, *p* = 0.47) was determined.

### EPH Conditioned Place Preference Already Occurs at Non-stimulatory Doses

DAT expression is known to play a role in the reward conditioning effects of psychostimulants such as cocaine (Medvedev et al., 2005); therefore, we investigated how male and female adolescent mice (*n* = 5–10) conditioned to either a non-locomotor stimulatory 5 or stimulatory 15 mg/kg dose of EPH. As evident by the increased time spent on the drug-paired side following eight sessions (four sessions each of vehicle or drug) of conditioning (Figure 5B), both the non-stimulatory (5 mg/kg) and stimulatory (15 mg/kg) dose of EPH increased time spent on the EPH-paired side, suggesting reward. A significant effect of conditioning (*F*_1,27_ = 88.9, *p* < 0.0001) was observed, while no effect of drug dose (*F*_3,27_ = 0.583, *p* = 0.63) or drug dose × conditioning (*F*_3,27_ = 1.15, *p* = 0.35) was noted by two-way ANOVA, with a significant effect of matching (*F*_27,27_ = 3.17, *p* = 0.0019). For adolescent male mice exposed to 5 mg/kg or 15 mg/kg EPH, a significant increase in time spent on the EPH-paired side was observed (*p* < 0.0001, *p* = 0.0001, respectively). This increased in time spent on the EPH-paired side was also observed in female adolescent mice at 5 mg/kg and 15 mg/kg as well (*p* = 0.0218, *p* < 0.0001, respectively). Three-way ANOVA with sex (S), drug treatment (D, 5 vs. 15 mg/kg) and time (T, pre vs. post) as factors did not find significant main effects for S × D (*F*_1,27_ = 0.169, *p* = 0.68), S × T (*F*_1,27_ = 0.018, *p* = 0.89), or S × D × T (*F*_1,27_ = 2.489, *p* = 0.126) (Figure 5B).

### EPH Dose-Dependently Increases ΔFosB Expression in Striatal and Cortical Areas

**Figure 6:**
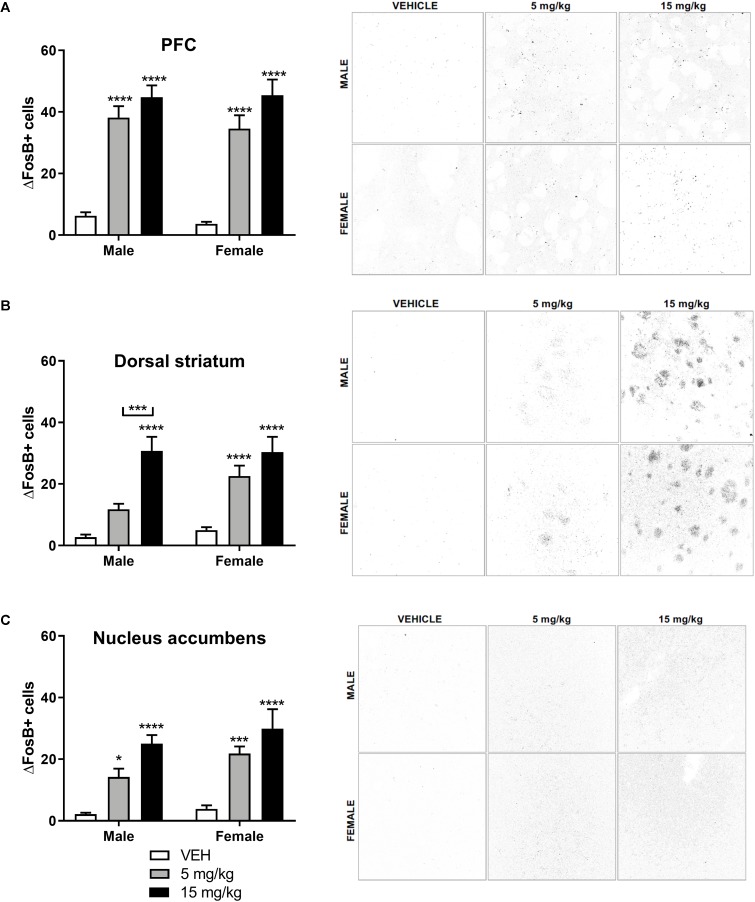
EPH exposure dose-dependently increases ΔFosB expression in striatal and cortical areas. Repeated systemic (i.p.) exposure to vehicle or EPH (5 or 15 mg/kg.) significantly increased ΔFosB expression in the prefrontal cortex **(A)**, dorsal striatum **(B)**, and nucleus accumbens **(C)** in both male and female adolescent mice. Dose-dependent increases in ΔFosB expression is observed in the dorsal striatum and nucleus accumbens, but not in the prefrontal cortex. Scale bar = 100 μm. Significance by two-way ANOVA with Tukey’s method for multiple comparisons, ^∗^*p* < 0.05; ^∗∗∗^*p* < 0.001; ^∗∗∗∗^*p* < 0.0001; data represented as mean ± SEM.

ΔFosB, a long-lasting neuronal marker heavily implicated in drug addiction (Kelz et al., 1999; Nestler et al., 2001; Perrotti et al., 2008), has been shown to increase upon repeated exposure to drugs of abuse in mesocorticolimbic brain regions (Nestler et al., 2001). Because we observed robust EPH induced CPP, we questioned whether EPH exposed mice would exhibit strong ΔFosB expression in mesocorticolimbic brain regions. We exposed male and female adolescent mice (*n* = 8–9) to vehicle, 5, or 15 mg/kg EPH (i.p.) once daily for 12 consecutive days to measure changes in ΔFosB in the brain. Three days after the final exposure, mice were sacrificed and brains were extracted. A significant increase in ΔFosB accumulation was observed in the prefrontal cortex (Figure 6A), dorsal striatum (Figure 6B), and nucleus accumbens (Figure 6C) in animals exposed to EPH. In the prefrontal cortex, a significant effect of drug dose (*F*_2,43_ = 74.4, *p* < 0.0001) was observed, with no sex (*F*_1,43_ = 0.418, *p* = 0.521) or interaction (*F*_2,43_ = 0.205, *p* = 0.816) effect, and multiple comparisons reveled that both 5 and 15 mg/kg significant increased ΔFosB staining in both male and female mice (*p* < 0.0001) as compared with vehicle. In the dorsal striatum, a significant effect of drug dose (*F*_2,43_ = 37.8, *p* < 0.0001) was observed, with no sex (*F*_1,43_ = 2.71, *p* = 0.107) or interaction (*F*_2,43_ = 1.63, *p* = 0.207) effect. Multiple comparisons revealed that 15 mg/kg EPH significant increased ΔFosB as compared with vehicle (*p* < 0.00001) in both male and female mice. In males, a significant increase was observed between 5 and 15 mg/kg (*p* = 0.0005), and in females, a significant increase was noted between vehicle and 5 mg/kg (*p* = 0.0006). For the nucleus accumbens, a significant effect of drug dose (*F*_2,43_ = 32.9, *p* < 0.0001) was observed, with no sex (*F*_1,43_ = 3.49, *p* = 0.0686) or interaction (*F*_2,43_ = 0.453, *p* = 0.639) effect, where multiple comparisons revealed that 15 mg/kg EPH significant increased ΔFosB as compared with vehicle (*p* < 0.00001) in both male and female mice. In the nucleus accumbens, a significant increase in ΔFosB in male and female mice exposed to 5 mg/kg compared with vehicle (*p* = 0.0265, *p* = 0.0004, respectively) was found.

## Discussion

Here, we assessed the cognitive and rewarding effects and neurochemical impact of repeated EPH exposure in both adolescent male and female C57Bl/6 mice. We specifically evaluated drug responses in this age group as adolescent reports of NPS use (and drug experimentation in general) are prevalent (Steinberg, 2008; Patrick et al., 2016). We observed that repeated exposure to 15 mg/kg EPH decreased spatial cognitive performance as assessed by the Barnes maze task in adolescent male mice, although this was not associated with a decrease in mature BDNF in any of the brain regions tested. EPH increased locomotor activity at 15 mg/kg, but not 5 mg/kg, and did not induce locomotor sensitization upon repeated exposure. Reward to EPH (as measured by CPP) was observed at both the non-locomotor stimulatory dose of 5 mg/kg and the stimulatory dose of 15 mg/kg EPH. Repeated EPH exposure dose-dependently correlated with increased ΔFosB expression in the dorsal and ventral striatum, while in the prefrontal cortex, both 5 and 15 mg/kg EPH significantly increased ΔFosB similarly with no difference in dose. Overall, our results suggest that EPH is indeed rewarding and stimulating as human reports would suggest (Ho et al., 2015; Soussan and Kjellgren, 2015), and although it causes spatial cognitive deficits at doses which cause hyperactivity, this did not seem to correlate with changes in proBDNF or mature BDNF expression. Importantly, no sex differences were observed between male and female animals throughout our testing. This was surprising as female rodents typically exhibit increased sensitivity to psychostimulants such as MPH (Roeding et al., 2014), cocaine (Lynch and Carroll, 1999), modafinil (Bernardi et al., 2015), and amphetamine (Van Swearingen et al., 2013) in behaviors associated with reward and drug self-administration.

Human self-reports suggest that EPH is consumed for its perceived cognitive enhancing effects (Ho et al., 2015; Soussan and Kjellgren, 2015), which is unsurprising given similar reports of misuse of ADHD medications such as amphetamine and MPH (Urban and Gao, 2017). In young adult mice, the cognitive benefits of 10 mg/kg MPH administered both pre-training and during training was shown to increase performance on spatial tasks upon repeated MPH exposure (Carmack et al., 2014a). This increase in performance was also observed in adult transgenic 5×FAD mice and Neurogranin knockout mice (Huang and Huang, 2012; Schneider et al., 2015), although the later study did not find a pro-cognitive effect of MPH in WT mice. Given the limited available data cognitive effects of MPH use in adolescent mice, we decided to assess whether EPH would affect spatial learning. We found that naïve male mice performed significantly better during the second training period compared to the first period, while female mice did not show an improvement, which is in line with previous studies (Bettis and Jacobs, 2009; O’Leary and Brown, 2013). Repeated EPH exposure attenuated the cognitive effects of repetition, but we did not see an effect of EPH exposure on female exposure (Figure 2B).

Increased hippocampal BDNF expression is commonly correlated with increased spatial task performance (Radecki et al., 2005; Petzold et al., 2015). Indeed, adolescent exposure to 10 mg/kg MPH increased BDNF expression in the dentate gyrus (Lee et al., 2012). However, the observed reduction in Barnes maze performance in male mice was not associated with a decrease in mature BDNF expression in male hippocampus (Figure 4). In rats, adolescent MPH exposure did not alter BDNF protein expression in adult PFC (Andersen and Sonntag, 2014). It is possible that the discrepancy in mature BDNF hippocampal expression versus Barnes maze performance in EPH-exposed animals arises from the timing of the experiments performed, as animals in the Barnes maze task were not administered drug prior to the training tasks, but rather in between pre-drug and post-drug exposure training sessions, and brains were collected 3 days after the last EPH exposure. An increase in proBDNF expression in the cortex and cerebellum was observed following EPH exposure in male adolescent mice (Figure 3); yet, no increase was observed in females following EPH exposure in these regions. It is possible that this sex differences in proBDNF levels in the cortex and cerebellum upon EPH exposure is correlated with the observed differences in Barnes maze performance follow EPH exposure (with males exposed to no drug recalling the task most effectively); however, because we did not run a time-course for proBDNF expression during the 12 daily EPH exposures and only measured expression 3 days after the final injection, it is unknown if proBDNF expression was higher during EPH exposure. No changes in mature BDNF levels were noted in the prefrontal cortex, in contrast to previous results in adolescent rats where repeated MPH exposure decreased mature BDNF expression in the prefrontal cortex (Scherer et al., 2010). It is unclear if this discrepancy is caused by experimental differences in drug (EPH vs. MPH), dose (10 vs. 15 mg/kg), or species (rats vs. mice). Additionally, levels of proBDNF and mature BDNF expression in mesolimbic regions such as the nucleus accumbens were not assessed here, although increases in BDNF gene expression in the ventral and dorsal striatum has been observed following MPH exposure in adolescent rats (Fumagalli et al., 2010).

In adolescent mice, EPH was stimulatory (Figure 1A) at a 15 mg/kg dose but not at 5 mg/kg. The tested doses of EPH chosen were based on those used for MPH in C57Bl/6 male mice (Carmack et al., 2014b; Heredia et al., 2014), where 10 mg/kg MPH is stimulatory and displays locomotor sensitization after 7 days of exposure (Carmack et al., 2014b) yet 1 mg/kg dose does not induce hyperlocomotion or locomotor sensitization in adult mice. Interestingly, we observed no locomotor sensitization to either 5 or 15 mg/kg EPH despite the 12 days of exposure (Figure 5A), although both doses were found to be rewarding in our CPP paradigm (Figure 5B). Carmack et al. (2014b) found that both a non-stimulatory and stimulatory MPH dose were rewarding as measured by CPP. Similarly, the DAT specific inhibitor GBR-12783 also produces CPP at non-stimulatory and stimulatory doses (Le Pen et al., 1996). Both these findings agree with our results for EPH and appear to be associated with DAT inhibition. Overall, these results suggest that the locomotor and reward profile of EPH at 5 and 15 mg/kg is similar to that observed at 1 and 10 mg/kg MPH despite the differences in DAT versus NET preference observed by *in vitro* transporter binding (Patrick et al., 2005; Williard et al., 2007).

Measuring BDNF quantification in mesolimbic regions following EPH would have been insightful as altered levels of BDNF are observed following cocaine exposure (Li and Wolf, 2015). However, as mesolimbic BDNF plays multiple roles in drug addiction, with alterations depending on brain subregion examined (nucleus accumbens core vs. shell) and timing in relation to drug exposure (withdrawal vs. intoxication) (Li et al., 2013), we chose to assess ΔFosB in these regions as it is a known stable (∼8 days) marker for neuronal activation and would be less sensitive to timing of last drug exposure (Nestler et al., 2001). Repeated exposure to EPH increased ΔFosB expression in both male and female adolescent mice in cortical and striatal regions of the brain (Figure 6), and this increase in the striatum is similar to the increase in fosB [a non-truncated splice variant of ΔFosB (Nestler et al., 2001)] immunoreactivity observed in adolescent male rats exposed to 2 or 10 mg/kg MPH for 14 days (Chase et al., 2005). As increased ΔFosB expression is associated with increased sensitivity to the behavioral effects of certain drugs of abuse (Nestler et al., 2001), both our results for EPH and previous reports for MPH would suggest that these drugs may have an abusive profile and/or alter future drug seeking behaviors. Increases in ΔFosB expression observed in our study are presumed to be the result of increased dopamine levels in brain regions following drug exposure (Nestler et al., 2001) as the result of DAT inhibition by EPH (Chase et al., 2005). Interestingly, a study by Cummins et al. (2013) found that repeated exposure to 5 mg/kg MPH in adolescence decreases DAT expression in the nucleus accumbens and striatum compared with vehicle control (Moll et al., 2001), suggesting that repeated drug exposure downregulates one of the molecular targets (DAT) of both MPH and EPH and importantly, decreased DAT expression would presumably increase extracellular dopamine levels by preventing dopamine reuptake. As this decrease in transporter expression upon MPH exposure in adolescent and adult rodents appears to be unique to DAT (as compared with NET or SERT) (Izenwasser et al., 1999; Moll et al., 2001), future studies may evaluate if EPH has a similar effect on DAT expression in the regions where increased ΔFosB expression was observed.

In summary, in this study we characterized the NPS EPH in relation to its self-reported effects in humans (Ho et al., 2015; Soussan and Kjellgren, 2015) on drug sensitization, reward, and cognition following repeated exposure in adolescent male and female C57Bl/6 mice. With findings of decreased cognitive performance, significant reward, and increased ΔFosB expression following prolonged, repeated EPH exposure, our animal models provide evidence that EPH is indeed stimulating and rewarding, and thus may have an abusive profile. As the current legal status of EPH in the United States is not explicitly clear, these determined behaviors in male and female adolescent mice suggest that EPH’s effects on behavior are similar to its similar chemical analog, MPH, a Schedule II substance. However, EPH use may have stronger negative aspects in terms of learning, but this will require further comparative studies with MPH to properly discern.

## Author Contributions

MR, ALB, and RvR conceptualized and designed the study, and critically edited and proofread the manuscript. MR and ATB did the acquisition of data. MR, ATB, JH, and RvR analyzed and interpreted the data. MR drafted the manuscript.

## Conflict of Interest Statement

The authors declare that the research was conducted in the absence of any commercial or financial relationships that could be construed as a potential conflict of interest.

## Abbreviations

BDNF: brain-derived neurotrophic factor
CPP: conditioned place preference
DAT: dopamine transporter
EPH: ethylphenidate
MPH: methylphenidate
NET: norepinephrine transporter
NPS: novel psychoactive substance
SERT: serotonin transporter.
